# Bioprospecting of Plant Growth Promoting Bacilli and Related Genera Prevalent in Soils of Pristine Sacred Groves: Biochemical and Molecular Approach

**DOI:** 10.1371/journal.pone.0152951

**Published:** 2016-04-25

**Authors:** Nathaniel A. Lyngwi, Macmillan Nongkhlaw, Debajit Kalita, Santa Ram Joshi

**Affiliations:** Microbiology Laboratory, Department of Biotechnology & Bioinformatics, North - Eastern Hill University, Shillong, Meghalaya, India; Loyola University Chicago, UNITED STATES

## Abstract

*Bacillus* spp. and related genera native to soils of the pristine sacred groves from Meghalaya, India were characterized using biochemical and 16S rRNA gene analysis which revealed dominance of *Bacillus*, *Paenibacillus*, *Lysinibacillus* and *Viridibacillus* in the groves. Biochemical estimation was carried out for *in vitro* testing of plant growth promoting traits present in these isolates. PCR screening were performed for plant growth-promoting related genes involved in the biosynthesis of acid phosphatase (*AcPho*), indolepyruvate decarboxylase (*ipdC*), 1-aminocyclopropane-1-carboxylate deaminase (*accd*) and siderophore biosynthesis protein (*asbA*). 76% of the sacred grove isolates gave an amplified fragment for *AcPho*. Three of the isolates gave an amplified fragment for *Ipd*C gene. Apart from 2 isolates, all the other isolates including the reference strains were positive for the amplification of the *accd* gene indicating their potential to produce ACC deaminase enzyme. 42% of the isolates gave an amplified fragment for *asbA* gene indicating the potential ability of these isolates to produce the catechol type siderophore, petrobactin. Overall findings indicated multiple PGP genetic traits present in these isolates which suggested that these isolates are capable of expressing multiple PGP traits. Phylogenetic and sequence analysis of *accd* and *asbA* genes from the isolates revealed that *asbA* genes from *Paenibacillus taichungiensis* SG3 and *Paenibacillus tylopili* SG24 indicated the occurrence of intergeneric horizontal transfer between *Paenibacillus* and *Bacillus*.

## Introduction

The state of Meghalaya, covering an area of 22 429km^2^ and located between 24°47’–26°10’ N and 89°45’–92°47’ E, is one of the species rich area under the mega biodiversity centers [1]. Ethnic people of the region are known to practice an age-old tradition of preserving primary forest patches near their settlements as part of their culture and religious belief. These primary forests called ‘sacred groves’, which are conserved due to the traditional religious beliefs not only constitute a major reservoir of the floral and faunal biodiversity, but are also assumed to be treasure troves of novel microorganisms [2].

Plant growth-promoting bacteria (PGPB) have direct stimulation on plant growth by providing plants with fixed nitrogen; soluble phosphates and nutrients; iron sequestered by bacterial siderophores; stimulate plant growth through the production of plant hormones such as indole-3-acetic acid (IAA) and activity of 1-aminocyclopropane-1-carboxylate deaminase (ACCD). Indirectly, these bacteria stimulate plant growth by inhibiting the growth of phytopathogenic microorganisms [3].

The genus *Bacillus* represents one of the most diverse genera in the class bacilli. It includes aerobic and facultatively anaerobic, rod-shaped, gram-positive spore-forming bacteria [4]. 16S rRNA gene sequence analysis has revealed a high level of phylogenetic heterogeneity in this genus, on the basis of which a division into different genera was proposed: *Bacillus*, *Alicyclobacillus*, *Paenibacillus*, *Brevibacillus*, *Aneurinibacillus*, *Virgibacillus*, *Solibacillus* and *Gracilibacillus* [5]. Here the term “*Bacillus* and related genera” is used as an operational term to indicate these organisms.

Genes responsible for PGP traits have been studied and explored intensely in recent years. In case of PGPB, several plant growth-promoting traits of these bacteria are a result of various genetic determinants that are present in their genome. With the advancement of metagenomic studies, information regarding the existence of genetic determinant conferring PGP traits has started to become clearer [6–8]. Several genetic determinants implicated in plant growth-promoting potential were investigated in the present study by considering the genes involved in the biosynthesis of acid phosphatase (*AcPho*; size ~ 734bp), indolepyruvate decarboxylase (*ipdC*; size ~ 1850bp), 1-aminocyclopropane-1-carboxylate deaminase (*accd*; size ~ 850bp) and siderophore biosynthesis protein (*asbA*; size ~ 1750bp) [9].

Mineralization of most organic phosphorus compounds is carried out by phosphatase enzymes and a significant amount of phosphatase activity in soil has been reported [10–13]. Major source of this activity in soil is considered to be of microbial origin [14,15]. Production of phytohormones like auxins is one of the direct mechanisms of enhancing plant growth by PGPB. Auxins like IAA have been shown to be produced by bacteria belonging to the genus *Bacillus* which help in stimulating plant growth [16]. In most bacteria, IAA is synthesized from the precursor tryptophan *via* the indole-3-pyruvic acid (IPyA) pathway where indole pyruvate decarboxylase (*ipdC*) is one to the most important enzyme in the pathway [17–19]. Another important mechanism by which PGPB stimulates the plant growth is through the activity of the enzyme ACC deaminase which causes lowering of plant ethylene levels resulting in longer roots. It catalyzes the cleavage of ACC, the immediate precursor of ethylene in plants, to α-ketobutyrate and ammonia thereby preventing/reducing the production of plant growth inhibiting levels of ethylene [20–21].

Microbial siderophores may stimulate plant growth directly by increasing the availability of iron in the soil surrounding the roots or indirectly by competitively inhibiting the growth of plant pathogens with less efficient iron-uptake system [22]. Many siderophores are small peptides synthesized by non-ribosomal peptide synthetases, which are multi-modular enzymes that produce peptide products with a particular sequence without an RNA template and can be chemically categorized as catecholates, hydroxamates, or α-hydroxy carboxylates based on their ferric iron ligand-binding functional groups [23]. One of the important siderophores secreted by *Bacillus* spp. is petrobactin, which is a catechol-type siderophore [24,25] and is synthesized *via* the action of six enzymes, encoded by the *asbABCDEF* gene cluster that includes *asbA*, *asbB*, *asbC*, *asbD*, *asbE* and *asbF* genes [26].

Horizontal gene transfer (HGT) refers to the movement of genetic material between species other than by descent in which information travels through the generations. HGT is known to enhance the survivability and proliferation of microbial communities in natural [27, 28]. It has been shown to be prevalent in the prokaryotic and bacterial genome with low frequencies of recombination and is considered to be responsible for generating diversity and adaptability among microorganisms [29]. While the role of HGT has been well documented for the transfer of antibiotic resistance, metal transporters and pathogenic genes in bacteria [30, 31], its contribution to the transfer of PGP related genes is scarce [32]. Phylogenetic incongruency between a gene of interest and a marker gene like 16S rRNA gene is often used as a stand-alone method for detecting HGT during gene evolution [33, 34]. Previously, plant growth promoting bacteria belonging to the genus ‘Bacillus and related genera’ were selectively isolated for exploring for PGP properties [35]. Preliminary screening for PGP traits have also been carried out for these isolates [35]. In the present study, bacterial isolates belonging to bacilli and related genera from the ‘Sacred Grove’ were explored for their plant growth promoting properties using biochemical and molecular approaches. The occurrence of HGT was also examined among the isolates of Bacillus and related genera by using the sequences of 16S rRNA gene and the PGP related genes.

## Materials and Methods

### Isolation and characterization of *Bacillus* and related genera

Soil samples were collected and analyzed from five different sacred groves of Meghalaya (24°47^/^–26°10^/^ N and 89°45^/^–92°47^/^ E) and bacterial isolation was performed as described earlier[1, 35]. For collection of soil samples, no permissions were required for these activities as the field studies did not involve endangered or protected species. The parameters observed for the isolates included microscopic appearance, spore-forming, gram stain, catalase test, oxidase test, and reduction of nitrate to nitrite [36]. *Bacillus* and related genera species were characterized by morphologies and physiology characteristics based on Bergeys’ Manual of Systematic Bacteriology [1, 2, 37].

### Biochemical characterization of PGP traits of the isolates

#### Phosphate solubilization

Isolates were qualitatively screened for phosphate solubilization on Pikovskaya’s agar plates [19]. The appearance of a transparent halozone around the colony indicated phosphate solubilizing activity of the isolate. Quantitative estimation of tricalcium phosphate {Ca 3 (PO 4) 2} solubilization in broth was carried out using Erlenmeyer flasks (250 ml) containing 100 ml of Pikovskaya’s broth inoculated with 1 ml of bacterial suspension (3 x 10 5 cells/ml). Un-inoculated controls were also used in each case. After 5 days incubation the bacterial culture was centrifuged at 5000 g for 15 min and the supernatant was used to estimate soluble phosphate (P) concentration. Broth P was determined by ammonium molybdate-ascorbic acid method [38]. The experiments were conducted in triplicates and data were expressed as the mean value ± standard error.

#### Siderophore production

Siderophore production was tested qualitatively following the method of Schwyn and Neilands[39]. Plates were incubated at 30°C and observed daily for yellow-orange color formation around each colony for 4 days. Catechol-type siderophores were measured in culture supernatants using Arnow’s assay [40], while hydroxamate siderophores were measured according to Csáky[41]. In the analyses, 2,3-dihydroxybenzoic acid and hydroxylamine hydrochloride, respectively, were used as standards. The experiments were conducted in triplicates and data were expressed as the mean value ± standard error.

#### Indole-3-acetic acid (IAA) production

Indole-3-acetic acid IAA production was analyzed using the method described by Wahyudi *et al*., where the presence of IAA was determined by the development of pink color[19]. IAA concentration was measured spectrophotometrically at 520nm and quantified in an IAA standard curve. The experiments were conducted in triplicates and data were expressed as the mean value ± standard error.

#### 1-aminocyclopropane-1-carboxylic acid (ACC) deaminase activity

ACC deaminase activity was screened adapting the method of Penrose and Glick [20]. Filter sterilized ACC solution (3mM) was spread over Dworkin and Foster (DF) minimal salts [42] agar plates and allowed to dry and inoculated with bacterial strains. Plates were incubated at 30°C for 3 days. The growth on the plates was checked daily. The ability of a strain to utilize ACC was verified by maintaining the same strain in a control in absence of nitrogen source. Based on the results from the first experiment, quantitative measurement of ACC-deaminase activity was carried out according the method of Penrose and Glick (2003)[20]. This method measures the amount of α-ketobutyrate (α-KB) produced when the enzyme ACC-deaminase cleaves ACC. The amount (mM) of α-KB produced by this reaction was determined by comparing the absorbance at 540 nm of a sample with a standard curve of α-KB. Experiments were conducted in triplicates and data were expressed as the mean value ± standard error.

#### Screening of PGP related genes

Genomic DNA was extracted using Genomic DNA isolation kit (HiPurA Bacterial and Yeast DNA Purification Spin Kit, HiMedia, India) and amplified using conditions and primer sets specific for different PGP related genes i.e., acid phosphatase (*AcPho*), indole pyruvate decarboxylase (*ipdC*), 1-aminocyclopropane-1-carboxylate deaminase (*accd*) and siderophore biosynthesis protein (*asbA*) as described by Raddadi *et al*. [9]. Amplified products were run on 1.2% agarose gels, stained with ethidium bromide and visualized under gel documentation system (UVItec, UK).

Amplicons of 1-aminocyclopropane-1-carboxylate deaminase (*accd*) and petrobactin biosynthesis protein (*asbA*) genes were purified using the QIAquick Gel Extraction Spin Kit (QIAGEN, Germany) and sequenced using the Big Dye Terminator cycle sequencing kitv.3.1 (Applied Biosystems, USA) deploying the standard protocol and an automated Genetic Analyzer ABI 3130XL (Applied Biosystems, USA).

### Phylogenetic analyses of 16S rRNA and *asbA* and *accd* genes

The 16S rRNA gene sequences of the isolates were searched for their homologue sequences in public domain databases. Basic Local Alignment Search Tool (BLAST) [43] was used to determine the phylogenetic neighbors from the nucleotide database of National Centre for Biotechnology Information (NCBI) and EzTaxon-e (the database of type strains with validly published prokaryotic names available online at http://eztaxon-e.ezbiocloud.net/) [44]. Basic Local Alignment Search Tool (BLAST, sub-program BLASTX) [43] was used to determine the phylogenetic neighbors of *asbA* genes from NCBI. Molecular Evolutionary Genetics Analysis software (MEGA v4.1) was used for phylogenetic analyses of 16S rRNA, *accd* and *asbA* genes. The obtained nucleotide sequences of identified phylogenetic neighbors were assembled and aligned using ClustalW inbuilt in MEGA 4.1 and phylogenetic tree was constructed using Neighbor-Joining method with 1000 bootstrap replications for nodal support [45]. The 16S rRNA gene sequence of *Serratia marcescens* AJ233431 was taken as an outlier. The G+C content of the sequenced *asbA* gene was calculated by using Oligo Calculator available at http://mcb.berkeley.edu/labs and was compared to the G+C contents of all other organisms belonging to the same genus.

## Results

### Isolation and characterization

Twenty six (26) bacterial isolates belonging to *Bacillus* and related genera were isolated as reported in Lyngwi et al. [35]. DNA sequencing and phylogenetic analysis revealed that the isolates showed 97–99% similarity to the sequences available in the NCBI GenBank. The 16S rDNA nucleotide partial sequences were submitted to GenBank and accession numbers from JX402416 to JX402441 was obtained for all the 26 sacred grove isolates (Table 1).

**Table 1:** PCR screening for PGP related genes from the isolates.

Isolates	Closest species (sequence similarity %)	*AcPho*	*IpdC*	*accd*	*asbA*
SG1	*Bacillus thuringiensis* (99.80) (JX402416)	+	–	+	+
SG2	*Lysinibacillus xylanilyticus* (100) (JX402417)	–	–	+	–
SG3	*Paenibacillus taichungensis* (99.93) (JX402418)	–	–	+	+
SG4	*Bacillus marisflavi* (99.80) JX402419	–	–	+	–
SG5	*Bacillus mycoides* (99.93) JX402420	+	–	+	+
SG6	*Bacillus thuringiensis* (98.92) JX402421	+	–	+	+
SG7	*Lysinibacillus parviboronicapiens* (99.12) JX402422	+	–	+	–
SG8	*Bacillus aryabhattai* (99.80) JX402423	+	–	–	–
SG9	*Bacillus safensis* (99.16) JX402424	–	–	+	–
SG10	*Bacillus cereus* (99.66) JX402425	+	+	+	+
SG11	*Bacillus thuringiensis* (99.93) JX402426	+	–	+	+
SG12	*Bacillus flexus* (100) JX402427	+	–	+	–
SG13	*Bacillus sonorensis* (99.50) JX402428	–	–	+	–
SG14	*Bacillus methylotrophicus* (99.65) JX402429	–	–	+	–
SG15	*Viridibacillus arenosi* (99.93) JX402430	+	–	+	–
SG16	*Bacillus psychrosaccharolyticus* (99.65) JX402431	+	–	+	–
SG17	*Bacillus thuringiensis* (99.73) JX402432	+	–	+	+
SG18	*Bacillus cereus* (99.73) JX402433	+	+	+	+
SG19	*Bacillus weihenstephanensis* (99.45) JX402434	+	–	+	+
SG20	*Bacillus mycoides* (100) JX402435	+	–	+	+
SG21	*Bacillus aryabhattai* (99.93) JX402436	+	–	–	–
SG22	*Bacillus humi* (98.30) JX402437	+	+	+	–
SG23	*Bacillus simplex* (97.29) JX402438	+	–	+	–
SG24	*Paenibacillus tylopili* (97.78) JX402439	+	–	+	+
SG26	*Viridibacillus arvi* (99.73) JX402440	+	–	+	–
SG27	*Bacillus methylotrophicus* (99.38) JX402441	+	–	+	–
	*Bacillus subtilis* MTCC 8141	+	–	+	–
	*Bacillus thuringiensis* MTCC 8996	+	+	+	+
	*Paenibacillus polymyxa* MTCC 9489	+	+	+	–
	*Bacillus cereus* MTCC10211	+	–	+	+

– indicates gene not amplified; + indicates gene amplified

### Biochemical estimation of plant growth promoting potential

**Fig 1:**
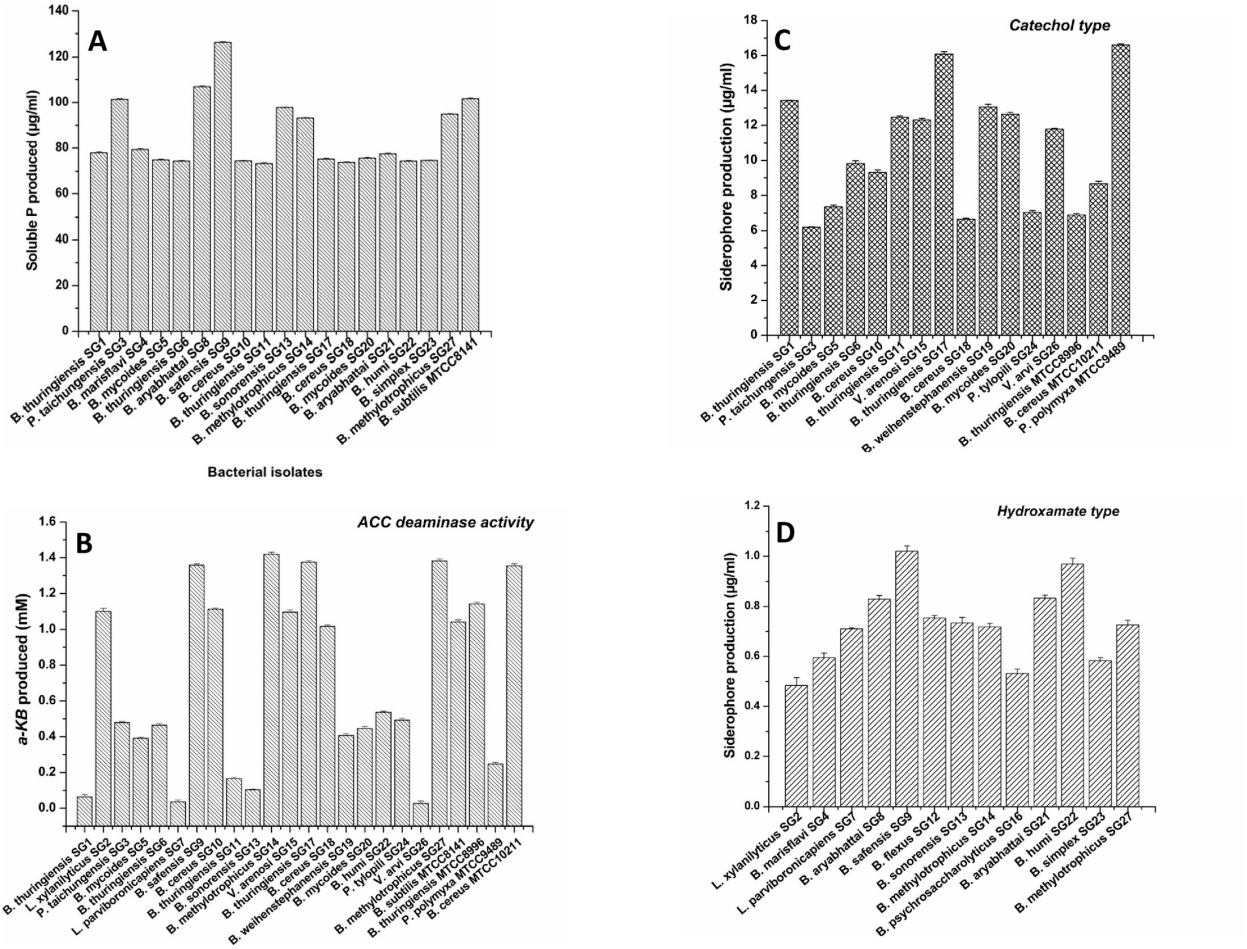
**Biochemical Estimation of PGP traits**: **A**) Soluble P production by the phosphate solubilizing isolates in liquid media; **B**) Catechol type siderophore production by the isolates; **C**) Hydroxamate type siderophore production by the isolates; **D**) IAA production by the different isolates. [Each value is mean of three replicates ± SE].

Previous qualitative studies with these isolates showed that they possess certain PGP traits that can be explored for further investigations [35]. Currently i*n vitro* studies were carried out to estimate the PGP properties in these isolates. Phosphorus (P) is major essential macronutrients for biological growth and development. Microorganisms offer a biological rescue system capable of solubilizing the insoluble inorganic phosphorus of soil and make it available to the plants. The ability of some microorganisms to convert insoluble phosphorus to an accessible form, like orthophosphate, is an important trait in a PGPB for increasing plant yields [46,47]. In this study, there was considerable variation among the isolates in the plant growth-promoting properties such as the phosphate solubilization and soluble P production by the phosphate solubilizing isolates (Fig 1A). The phosphate solubilization process on Pikovskaya’s agar was observed as solubilization haloes around the colonies. Based on the screening on Pikovskaya’s agar, 18(69%) of the sacred grove isolates were positive for phosphate solubilization. The concentration of soluble P in broth ranged between 73.33–126.23 μg/ml. The highest concentration of soluble P in the broth was detected for *Bacillus safensis* SG9 (126.23 μg/ml), while the lowest concentration was that of *B*. *thuringiensis* SG11 (73.33 μg/ml) (Fig 1A). The ability to solubilize phosphate was not detected in the isolates belonging to the genera *Lysinibacillus* and *Viridibacillus*. Another important trait of PGPB, that may directly or indirectly influence the plant growth, is the production of siderophore which is one of the most important attributes for biocontrol mechanisms of PGPB including bacilli groups [25]. All the characterized isolates in the present study showed the production of siderophores. Similar to phosphate solubilization, considerable variations were observed among the isolates in siderophore production and the quantity produced by the isolates (Fig 1B and 1C). Among all the isolates, the largest halozone for siderophore detection on CAS agar medium was formed by *Bacillus methylotrophicus* SG27 (8 ± 0.5 mm). Quantitative estimation of the amount of siderophore produced by the sacred grove isolates revealed that *B*. *thuringiensis* SG17 was the highest catechol-type siderophore producer (16.08 μg/ml) (Fig 1B) and *B*. *safensis* SG9 was the highest producer of hydroxamate-type siderophore (1.01 μg/ml)(Fig 1C). Interestingly, each isolate could produce only one type of siderophore but not both.

IAA (indole-3-acetic acid) is a member of the group of phytohormones and is generally considered the most important native auxin [3]. Among the PGP traits, indolic compounds such as IAA have a positive effect on root growth and morphology, and are thus believed to increase access to more nutrients in the soil [48]. All the studied isolates were found to produce Indole-3-acetic acid (IAA) but the amount of IAA production as determined in the culture supernatant differed among the isolates (Fig 1D). The concentration of IAA produced by the isolates in liquid medium ranged from 2.68–14.48 μg/ml. *Viridibacillus arenosi* SG15 (14.48 μg/ml) was the highest producer of IAA while *Bacillus humi* SG22 (2.68 μg/ml) was the lowest producer of IAA from among the characterized isolates.

Majority of the isolates showed ACC metabolism capacity. By using the ACC metabolism plate assay which is based on the ability of the isolates to use ACC as the sole nitrogen source through the action of ACC deaminase enzyme, it was observed that 20 (77%) of the sacred grove isolates were positive for ACC deaminase activity (Fig 1D). ACC deaminase production by the isolates varied considerably among the isolates. The amount of α-ketobutyrate produced by the isolates from ACC substrate, which indirectly indicated ACC deaminase production and activity, ranged between 0.03–1.42 mM, with *Bacillus methylotrophicus* SG14 (1.42 mM) and *Viridibacillus arvi* SG26 (0.03 mM) being the highest and lowest producer of α-ketobutyrate respectively (Fig 1D).

### Molecular characterization of plant growth promoting genes

Genes contributing to PGP traits were screened using primers that were described in Raddadi *et al*. [9]. Twenty (76%) of the sacred grove isolates gave an amplified fragment of the expected size for the *AcPho* gene. All the four MTCC reference strains were PCR-positive for this gene (S1A Fig; Table 1). Only 3 of the sacred grove isolates and 2 reference strains gave an amplified fragment of the expected size for the *Ipd*C gene (S1C Fig; Table 1). Apart from 2 isolates i.e, *Bacillus aryabhattai* (SG8 & SG21), all the other isolates including the reference strains were positive for *accd* gene (S1B Fig; Table 1). 11 (42%) of the sacred grove isolates and 2 reference strains gave an amplified fragment of the expected size for the *asbA* gene indicating the potential ability of these isolates to produce the catechol type siderophore, petrobactin (S1D Fig; Table 1). Overall in the present study, 2 sacred groves isolates namely, *Bacillus cereus* SG10 and *Bacillus cereus* SG18, and one reference strain *Bacillus thuringiensis* MTCC 8996 gave amplified fragments of the expected size for all the genes screened. On the other hand, 9 sacred groves isolates and 2 reference strains gave amplified fragments for 3 of the total 4 genes that were screened (Table 1). Nucleotide sequences of 1-aminocyclopropane-1-carboxylate deaminase (*accd*) and petrobactin biosynthesis protein (*asbA*) encoding genes have been deposited in NCBI with accession numbers assigned from KF874290 –KF874309 and KF874310 –KF874320 respectively.

### Horizontal Gene Transfer

Occurrence of phylogenetic incongruence between a marker gene like 16S rRNA gene and a gene of interest can be an indicator of horizontal gene transfer. Current studies, compares the phylogenetic trees constructed using 16S rRNA, *accd* and *asbA* genes respectively. BLASTX analysis was performed using the gene sequences of putative *accd* and *asbA* amplicons. The *accd* gene from these isolates shows high sequence similarity percentage with the *accd* genes of their respective homologs from the same genus and hence incidence of HGT was excluded amongst the isolates. However, out of the 11(42%) isolates that are positive for *asbA* gene, two isolates which were identified as *Paenibacillus* sp, i.e. *P*. *taichungiensis* SG3 and *P*. *tylopili* SG24 showed 98% sequence similarity with the *asbA* sequence of other genus (i.e. *Bacillus thuringiensis*) and displayed a poor sequence similarity percentage (<70%) when compared to the corresponding *asbA* sequence of the same genus (*Paenibacillus mucilaginosus* KNP414) reported in the GenBank (Table 2).

**Table 2:** Comparative matches for the closest phylogenetic neighbours obtained for the isolates based on profile of 16S rRNA gene and petrobactin biosynthesis protein *asbA* gene.

Isolates	Closest match of 16S rRNA with similarity percentage	Closest match of *asbA* rRNA with similarity percentage
SG1	*Bacillus thuringiensis*, 99.80%	*Bacillus thuringiensis*,98%
SG3	*Paenibacillus taichungiensis*, 99.93%	*Bacillus thuringiensis*, 98% *Paenibacillus mucilaginosus*, KNP414,70%
SG5	*Bacillus mycoides*, 99.93%	*Bacillus cereus*, 98%
SG6	*Bacillus thuringiensis*, 98.92%	*Bacillus thuringiensis*, 99%
SG10	*Bacillus cereus*, 99.66%	*Bacillus cereus*, 99%
SG11	*Bacillus thuringiensis*, 99.93%	*Bacillus thuringiensis*, 99%
SG17	*Bacillus thuringiensis*, 99.73%	*Bacillus thuringiensis*, 99%
SG18	*Bacillus cereus*, 99.73%	*Bacillus cereus*, 99%
SG19	*Bacillus weihenstephanensis*, 99.45%	*Bacillus cereus*, 100%
SG20	*Bacillus mycoides*, 100%	*Bacillus cereus*, 96%
SG24	*Paenibacillus tylopili*, 97.78%	*Bacillus thuringiensis*, 98% *Paenibacillus mucilaginosus*, KNP414,70%

**Fig 2:**
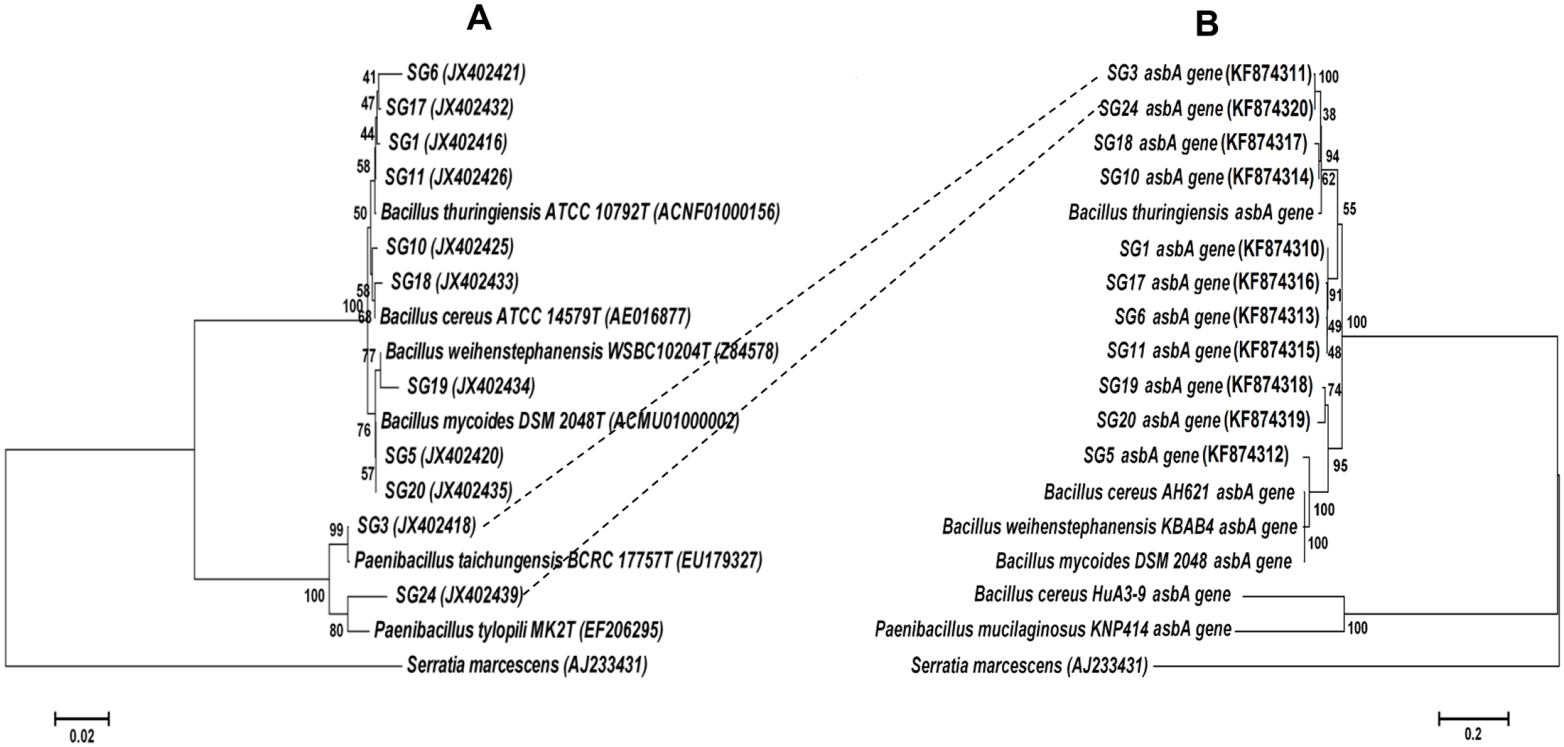
Phylogenetic incongruency between *asbA* and 16S rRNA. The genes encoding (A) 16S rRNA and (B) *asbA* protein of the isolates were subjected to neighbor-joining analysis. *asbA* positive isolates predicted to have undergone HGT are connected by dotted lines. Respective accession numbers of gene nucleotide sequences are indicated in bracket.

Phylogenetic analyses using Neighbor-Joining method were performed using the 16S rRNA gene sequence and also the *asbA* sequence of the 11 isolates (Fig 2). The nine *Bacillus* isolates clustered together with their corresponding genus in both the trees constructed using 16S rRNA gene sequence and *asbA* sequence. However, for the two isolates *Paenibacillus taichungiensis* SG3 and *Paenibacillus tylopili* SG24, their *asbA* gene sequences clustered together with the *asbA* sequence cluster of *Bacillus* genus unlike when their 16S rRNA gene sequence which clustered together with their corresponding *Paenibacillus* genus (Fig 2B, Table 2).

Furthermore, when another important criterion for implicating HGT, i.e., moles percent (mol%) of G+C content (atypical sequence composition) [49] was analyzed for the *asbA* sequences from *Paenibacillus taichungiensis* SG3 and *Paenibacillus tylopili* SG24, it was found that their G+C content (~35 mol%) is similar to that of *Bacillus thuringiensis* whole genome (32~35 mol%) whereas it is clearly lower than the G+C content reported for *Paenibacillus taichungiensis* (~46.7 mol%) [50]. Based on these criterion for implicating HGT, it can be said that the results obtained on analysis of the *asbA* sequence of *Paenibacillus taichungiensis* SG3 and *Paenibacillus tylopili* SG24, occurrence of HGT between *Paenibacillus* and *Bacillus* genus with respect to the siderophore biosynthesis protein (*asbA*) gene might have occurred during evolution of these co-existing and related groups of bacteria.

## Discussion

‘Sacred groves’ are pristine habitats that are inhabited by diverse groups of microorganism [35, 51]. Bacterial communities’ structures in these environments are influenced by the existing soil type and vegetation [52]. On the other hand, microbes also affect the soil component which in turn directly or indirectly impact the plant growth in that habitat [53]. Soil contains a mixture of both inorganic and organic source of nutrient that are either readily available to the plants or that required to be transformed into soluble form that can be taken up by the plants [54]. PGPB must have the ability to solubilize both mineral and organic nutrients and make it accessible to plants for their growth and development [53, 54]. Macronutrient like phosphorous and nitrogen are utilized by the plants in solubilized form [55]. Phosphate solubilization by PGPB can be achieved through the release of organic acids that chelates the insoluble phosphate through the action of phosphatase enzymes [55]. Eighteen of the isolates (69%) showed phosphate solubilizing properties indicating their ability to produce organic acids and/or phosphatase enzymes capable of converting insoluble phosphate to bioavailable phosphate (eg. orthosphosphate). Furthermore, 20 of the isolates showed the presence of acid phosphatase (*AcPho*) gene which could play a role in phosphate solubilization. Acid phosphatases play a major role in the mineralization of organic phosphorous in soil [56] and the presence of acid phosphatase (*AcPho*) gene indicate their potential role in mineralization of organic phosphorous in soil. One of the key mechanisms that PGPB use to support plant growth is through the production of phytohormones like auxins such as IAA [55, 57, 58]. All the isolates under study showed their ability to produce IAA even though the level of production varied as evident from the biochemical estimations. In bacteria, two pathways have been characterized for production of IAA: the indole-3-acetamide (IAM) pathway (L-tryptophan → IAM → IAA) and the indole-3-pyruvic acid (IPyA) pathway (L-tryptophan → IPyA → indole-3-acetaldehyde → IAA) [57]. In this study, the *IpdC* gene was chosen as the target because it has been reported that beneficial plant associated bacteria frequently synthesized IAA *via* the IPyA pathway [18]. The *IpdC* gene was detected in only 3 of the sacred grove isolates indicating their ability to synthesize IAA *via* the IPyA pathway and their potential as beneficial plant associated bacteria. Interestingly, most of the isolates that responded positively to the Salkowski’s test for IAA production (data not shown) did not give amplification for the *ipdC* gene. This could possibly be that the IAA production in these isolates is *via* the indole-3-acetamide (IAM) pathway and not the IPyA pathway [59].

Plant tends to synthesize ethylene in response to stress conditions and if present in high concentrations, can lead to growth inhibition and death [60]. ACC deaminases have been shown to protect plants from the deleterious effects of various biotic and abiotic stresses by lowering the inhibiting levels of ethylene in plants [61–65]. This enzyme catalyzes the breakdown of ACC which is the precursor of ethylene into ammonia and ∞-ketobutyrate [66]. Seventy seven percent of the isolates were able to metabolize ACC when used as sole nitrogen source indicating the presence of ACC deaminase enzyme. Moreover, almost all sacred grove isolates, except two isolates of *Bacillus aryabhattai* (SG8 & SG21), were positive for the amplification of the *accd* gene. PGPB expressing ACC deaminase can help in preventing the buildup of ethylene through an intricate and well regulated mechanism that balances the concentration of ethylene and its precursor (ACC) with minimal damage to the plants [21]. This high frequency presence of the *accd* gene could also be explained by considering that this enzyme could be implicated in the deamination of substrates other than ACC as was found for the ACCD enzymes from *Pseudomonas putida* UW4 [67] and *Pyrococcus horikoshii* [68].

Overall, 2 sacred groves isolates *viz*. *Bacillus cereus* SG10 and *Bacillus cereus* SG18, and one reference strain *Bacillus thuringiensis* MTCC 8996 gave amplified fragments of the expected size for all the PGP genes screened. On the other hand, 9 sacred groves isolates and 2 reference strains gave amplified fragments for 3 of the 4 genes that were screened. This indicated multiple PGP genetic traits that are present in these isolates which suggested that these isolates are capable of expressing traits that are important in biofertilization, biostimulation, bioprotection and biocontrol activities.

In the present study, the possible occurrence of HGT with respect to the siderophore biosynthesis protein (*asbA*) gene was observed among the two sacred groves isolates *Paenibacillus taichungiensis* SG3 and *Paenibacillus tylopili* SG24. The frequencies of HGT between closely related genera are comparatively higher [29] although with very low detection even at molecular level [69]. The high sequence similarity (98%) and almost similar G+C content (~35 mol%) between the *asbA* sequences of these two isolates with that of *Bacillus thuringiensis* instead of *Paenibacillus* sp. suggests that HGT might have occurred from *Bacillus* sp. to the *Paenibacillus* sp. since HGT creates an unusually high degree of similarity between the donor and the recipient strains for the character in question [70]. This was further supported by the phylogenetic incongruence that was observed in these two isolates. Although, the contribution of HGT to the transfer of PGP related genes is being reported with respect to ACC deaminase gene [7,32], this study reports for the first time the possible occurrence of HGT among bacilli isolated from sacred grove with respect to the siderophore biosynthesis protein (*asbA*) gene. The current study showed that bacterial isolates belonging to ‘*Bacillus* and related genera’ possess promising plant growth promoting properties that can be further explored for agrobiotechnological applications.

## Supporting Information

S1 Fig PCR products on agarose gel showing. **A**) *AcPho* gene; **B**) *ipdC* gene; **C**) *accd* gene;**D** *asbA*gene.
(TIF)
