# Supplementary figures and images for: Bioprospecting of Plant Growth Promoting Bacilli and Related Genera Prevalent in Soils of Pristine Sacred Groves: Biochemical and Molecular Approach
Source: PLoS One. 2016 Apr 25;11(4):e0152951. doi: 10.1371/journal.pone.0152951 (PMC4844137; doi:10.1371/journal.pone.0152951)

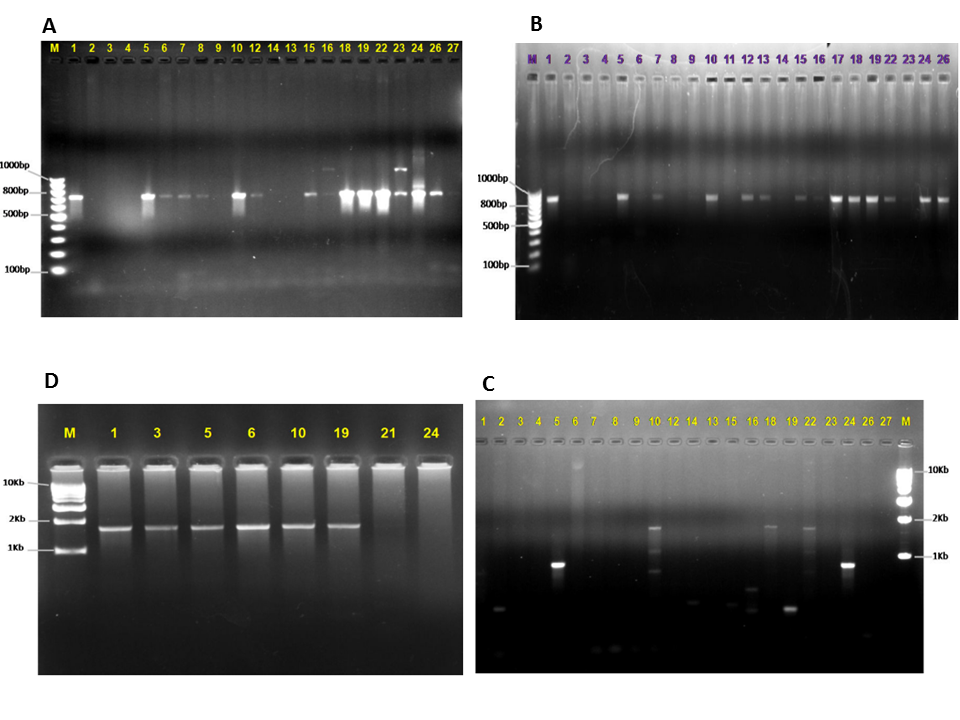

Supplement: S1 Fig — A) AcPho gene; B) ipdC gene; C) accd gene;D asbAgene. (TIF) [file pone.0152951.s001.tif]
